# Osh2 mediates *Candida* species resistance to miltefosine by regulating zymosterol accumulation

**DOI:** 10.1128/aac.00427-25

**Published:** 2025-07-23

**Authors:** Yongqin Wu, Yuanyuan Dai, Huaiwei Lu, Xiaohua Jiang, Yuanyuan Wang

**Affiliations:** 1 Department of Laboratory Medicine, The First Affiliated Hospital of USTC, Division of Life Sciences and Medicine, University of Science and Technology of China 12652, Hefei, Anhui, China; 2 Core Unit of National Clinical Research Center for Laboratory Medicine, Hefei, Anhui, China; 3 The Center for Reproduction and Genetics, Department of Obstetrics and Gynecology, The First Affiliated Hospital of USTC, Division of Life Sciences and Medicine, University of Science and Technology of China 12652, Hefei, Anhui, China; 4 The Center for Microbes, Development, and Health, Key Laboratory of Molecular Virology and Immunology, Unit of Pathogenic Fungal Infection & Host Immunity, Shanghai Institute of Immunity and Infection, Chinese Academy of Sciences 85402 , Shanghai, China; University Children's Hospital Münster, Münster, Germany

**Keywords:** miltefosine, *Candida *species, Osh2, zymosterol, resistance

## Abstract

Invasive candidiasis poses a growing threat to global public health, compounded by the scarcity of effective antifungal treatments. Miltefosine exhibits broad-spectrum antifungal activity, yet its mechanisms of antifungal action and the development of resistance remain poorly understood. Here, we first generated miltefosine-resistant strains of *Candida glabrata* through stepwise exposure to increasing drug concentrations. Whole-genome sequencing revealed that nonsense mutations in the *OSH2* gene (193C > T and 3177C > A) were key drivers of resistance. Functional validation in *Candida albicans* confirmed that these *OSH2* mutations conferred miltefosine resistance, demonstrating the conserved role of Osh2 across species. Multi-omics profiling of the *osh2Δ/Δ* mutant revealed significant upregulation of ergosterol biosynthesis genes, including *ERG6* and *ERG11*, and the accumulation of zymosterol, an intermediate in the ergosterol pathway. Chemogenetic dissection further elucidated the role of sterol metabolism in resistance: *erg11Δ/Δ* mutants, which are unable to synthesize zymosterol, exhibited hypersusceptibility to miltefosine, whereas *erg6Δ/Δ* strains, which accumulate zymosterol, showed innate resistance. Exogenous supplementation of zymosterol dose dependently increased the minimum inhibitory concentration of miltefosine in *C. albicans* and *C. glabrata*, confirming that zymosterol accumulation is a key determinant of resistance. Our findings establish Osh2 as a critical regulator of membrane sterol flux and demonstrate that fungal lipid metabolic plasticity enables evasion of membrane-targeting antifungals. Therapeutic targeting of zymosterol biosynthesis enzymes may overcome such adaptive resistance mechanisms in invasive candidiasis, providing a new strategy to combat drug-resistant fungal infections.

## INTRODUCTION

Invasive candidiasis represents a significant threat to global healthcare, particularly in hospital settings, with over 1.5 million cases of *Candida* bloodstream infections or invasive candidiasis estimated annually (1, 2). With mortality rates reaching 63.6% in high-risk populations and multidrug-resistant strains emerging across major *Candida* species (*C. auris*, *C. glabrata*), in particular, *C. albicans* and *C. auris* have been classified by the World Health Organization as critical priority pathogens requiring urgent therapeutic innovation (1–3). Although antifungal agents such as azoles, polyenes, and echinocandins are available, their clinical utility is significantly limited by toxicity and resistance (4). Miltefosine, licensed for treating human and canine leishmaniasis, has emerged as a promising broad-spectrum antifungal agent (5–8). Originally developed as an anticancer drug, miltefosine has demonstrated potent activity against a wide range of fungal pathogens, including *Candida*, *Cryptococcus*, *Aspergillus*, *Fusarium*, *Scedosporium*, *Lomentospora*, and *Mucorales* species (9–15). Notably, miltefosine has been granted orphan drug designation by the U.S. Food and Drug Administration for the treatment of invasive *Candida* infections, highlighting its potential as a novel therapeutic option (16).

The antifungal activity of miltefosine is attributed to its multifaceted mechanisms of action, which include disrupting fungal cell membrane integrity, inducing oxidative stress, and triggering apoptotic pathways (17–19). Studies have shown that miltefosine interferes with sphingolipid metabolism, leading to the leakage of intracellular components and disorganization of lipid rafts (10, 11, 20). Additionally, the exogenous addition of glucosylceramide and ergosterol (a key component of fungal membranes) reduces miltefosine’s inhibitory activity, highlighting the importance of membrane lipid composition in its mechanism of action (10, 11). The capacity of miltefosine to target multiple cellular processes renders it a highly potent antifungal agent, yet this multiplicity also highlights the complexity of its mechanism of action and the potential for a diverse array of resistance mechanisms to emerge. Recent studies have shown that resistance to miltefosine in *C. albicans* and *C. parapsilosis* is associated with increased expression of the *RTA3* gene (16, 21). However, the mechanisms underlying miltefosine resistance in fungi remain poorly understood.

Drug target mutations are a well-documented mechanism of antifungal resistance in *Candida* species. For instance, mutations in the *ERG11* gene, which encodes lanosterol demethylase, confer resistance to azoles by reducing drug-binding affinity (22, 23). Similarly, mutations in *FKS1*, the target of echinocandins, lead to reduced susceptibility by altering the drug’s interaction with β-1,3-glucan synthase (24, 25). These examples highlight that inducing resistance in laboratory strains and identifying associated mutations can provide critical insights into the molecular targets and mechanisms of action of antifungal drugs (26). However, to date, no laboratory-evolved miltefosine-resistant strains have been reported. Inducing resistance to miltefosine not only serves as a method to evaluate its potential for sustained clinical use but also offers a powerful approach to uncover its antifungal mechanisms and identify potential drug targets.

In this study, we attempted to induce miltefosine resistance in various *Candida* species using a gradient concentration induction approach. While this method successfully generated resistant strains in *C. glabrata*, attempts in other *Candida* species were unsuccessful. This highlights the low propensity of miltefosine to induce resistance and suggests its potential for sustained clinical use. Through a combination of whole-genome sequencing and functional genomics, we identified key genetic and metabolic changes associated with miltefosine resistance. Our findings reveal that nonsense mutations in the oxysterol-binding protein-coding gene *OSH2*, which regulates zymosterol transport, play a critical role in mediating resistance through the accumulation of zymosterol and modulation of the ergosterol biosynthesis pathway. This study is the first to demonstrate the role of Osh2 in miltefosine resistance, highlighting its function in sterol metabolism and its impact on antifungal drug susceptibility. These insights not only advance our understanding of miltefosine’s mode of action but also provide a foundation for developing novel strategies to combat antifungal resistance.

## RESULTS

### Loss-of-function mutations of Osh2 confer resistance to miltefosine

**Fig 1:**
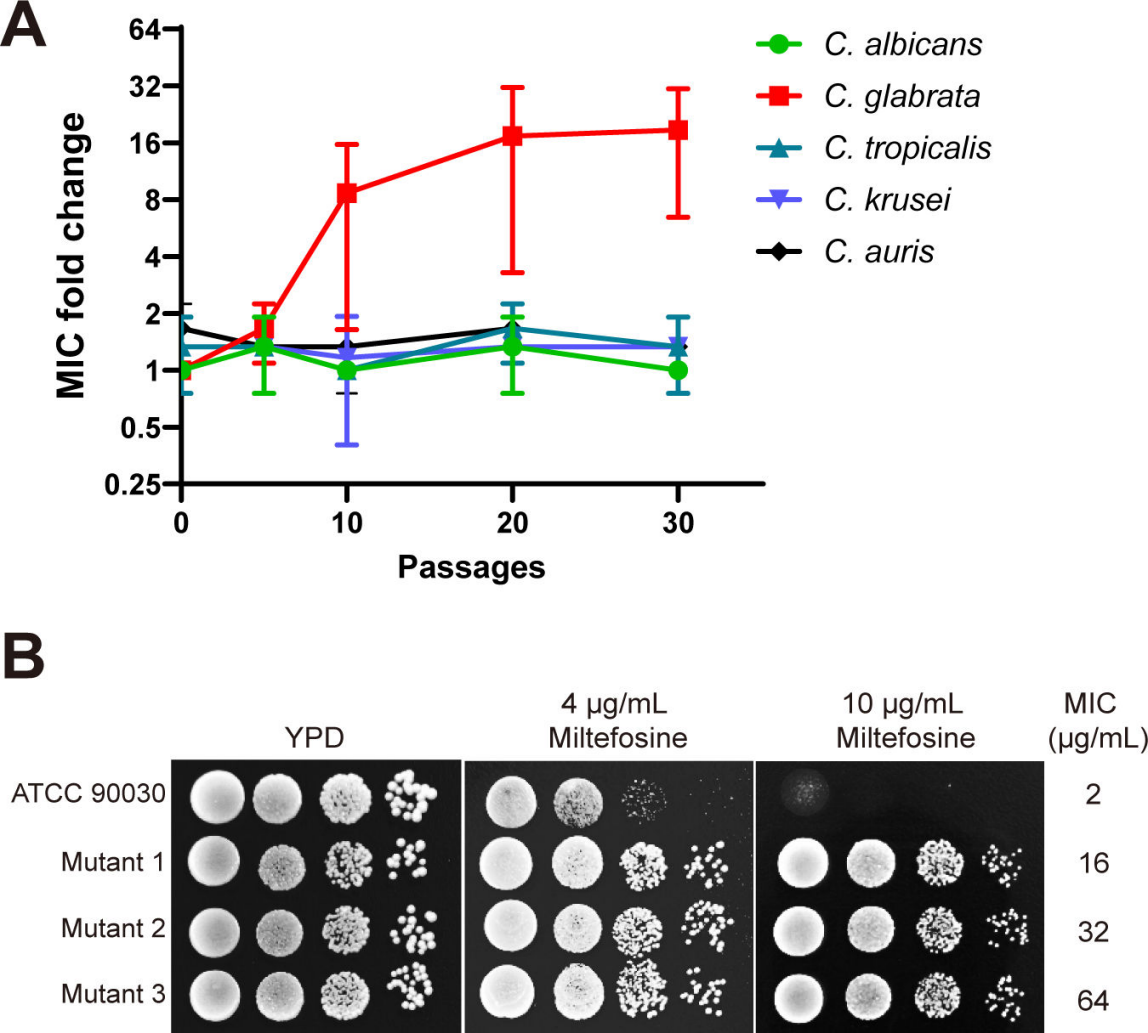
Generation of miltefosine-resistant strains through the concentration gradient induction of drug resistance. (**A**) The minimum inhibitory concentrations of miltefosine were measured for *Candida albicans* SC5314, *Candida glabrata* ATCC90030, *Candida tropicalis* ATCC750, *Candida krusei* ATCC6258, and *Candida auris* CBS12766. Yeast cells of these strains that grew at 0.5× MIC of miltefosine were first cultured without miltefosine, then recultured in its presence. This process counts as a single passage; only after the yeast develops tolerance to this concentration of miltefosine can higher concentrations be applied. After different passages, the yeasts were recovered for microbroth dilution MIC testing. The fold increase in MIC of the mutants compared to the parent strains was determined. Three biological replicates and 30 passages were performed. (**B**) Verification of miltefosine resistance in three independent mutant strains (mutant 1, mutant 2, and mutant 3) derived from parallel laboratory evolution experiments was conducted using the spot growth assay and microbroth dilution MIC testing.

To obtain laboratory-evolved miltefosine-resistant *Candida* strains, we employed a stepwise increase in drug concentration to induce resistance in various *Candida* species. After 30 rounds of induction, only *C. glabrata* ATCC90030 successfully mutated, with the mutants’' minimum inhibitory concentrations (MICs) for miltefosine increasing 8- to 32-fold compared to the parental strain (Fig. 1A). Spot growth assays were performed on three independent mutant strains (mutant 1, mutant 2, and mutant 3) derived from parallel laboratory evolution experiments, all demonstrating significant resistance to miltefosine (Fig. 1B). These results indicate that the miltefosine-resistant strains were successfully established. Additionally, they suggest that *Candida* species generally exhibit a lower propensity to develop resistance to miltefosine.

**Fig 2:**
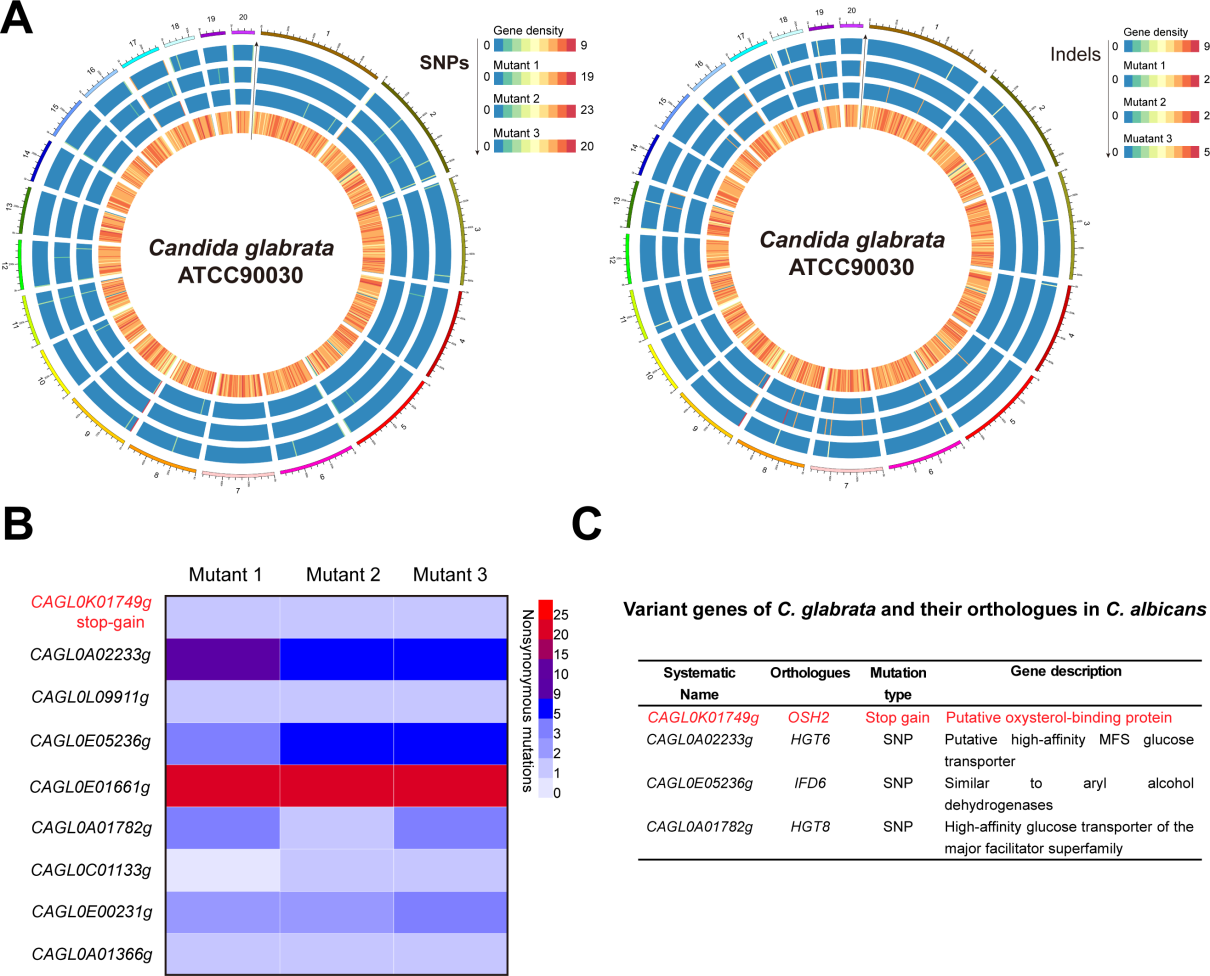
Gene mutations associated with miltefosine resistance were identified through whole-genome sequencing. (**A**) Using the R package circlize, we create circular plots to show whole-genome SNPs (left panel) and Indels (right panel) variation data. The innermost ring displays the gene density of the reference genome of *C. glabrata* ATCC90030. Surrounding the innermost ring, the second through fourth circles sequentially represent the genomic variation profiles of the evolved mutants. The depth of color in these rings indicates the amount of genetic variation. The outermost ring offers an overview of the size of the reference genome. (**B**) Gene missense mutation profile of three miltefosine-resistant mutants. (**C**) Functional annotations and orthologs of the variant genes in *C. albicans*.

To further explore the molecular mechanisms underlying the development of resistance to miltefosine in *C. glabrata*, we performed *de novo* whole-genome sequencing on *C. glabrata* ATCC90030 and resequenced the same three mutant strains (mutant 1 to mutant 3) described above. The analysis identified some single-nucleotide polymorphisms (SNPs) and insertions/deletions (Indels) across the mutant strains (Fig. 2A). However, missense mutations were predominantly clustered in nine genes, including *CAGL0K01749g*, which harbored two premature stop codon mutations (193C > T and 3177C > A) (Fig. 2B; Table S1). The nonsense mutations of the *CAGL0K01749g* gene were additionally confirmed by bidirectional Sanger sequencing (Fig. S1). The gene *CAGL0K01749g* shares identity with the *OSH2* gene in *C. albicans*. Other mutated genes identified in *C. albicans* include *HGT6*, *IFD6*, and *HGT8* (Fig. 2C). These mutations are likely to be implicated in the development of miltefosine resistance in *C. glabrata*.

**Fig 3:**
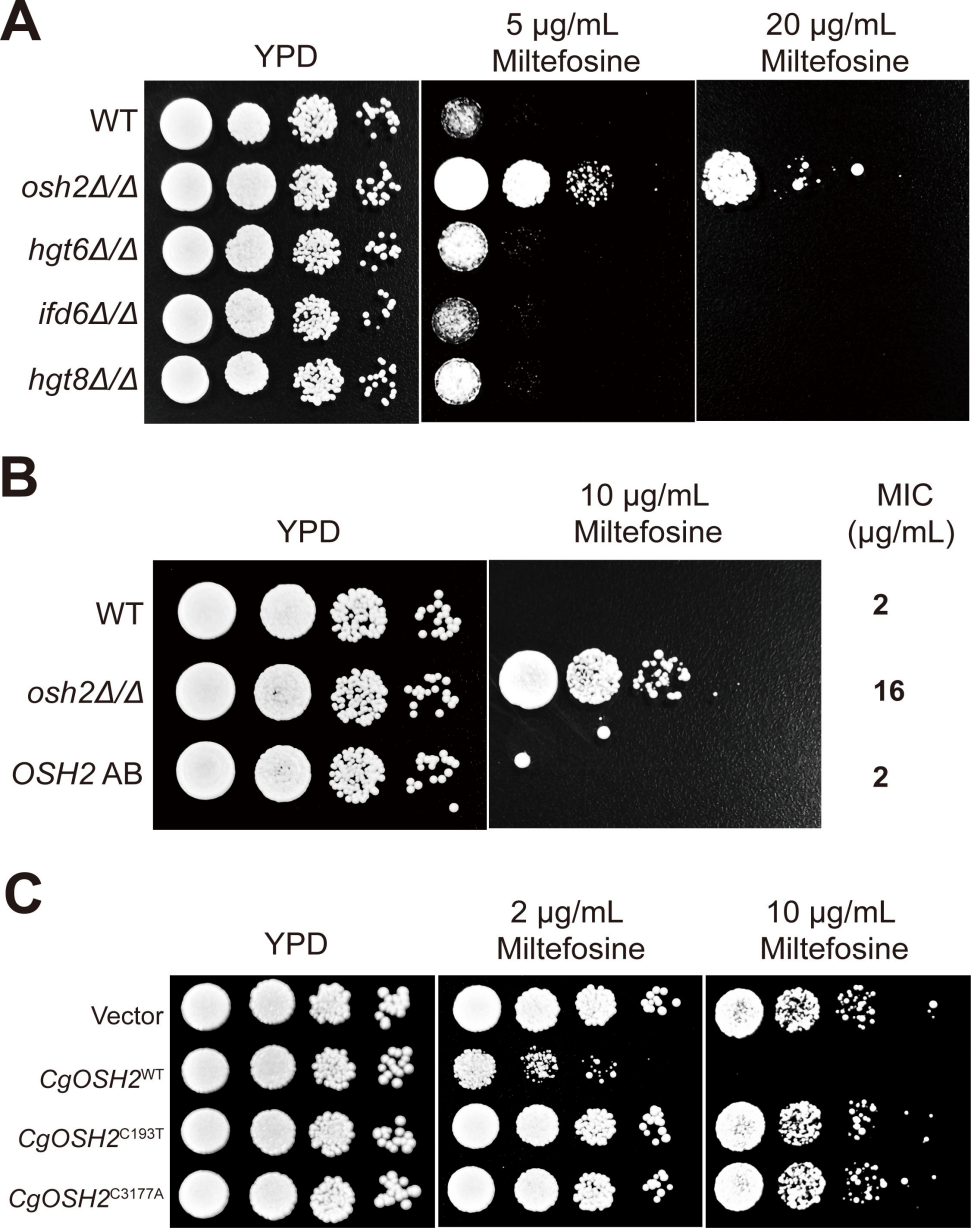
Loss-of-function mutations of Osh2 confer resistance to miltefosine. Spot growth assays were performed on various gene knockout mutants of *C. albicans* (**A**), osh2 mutants (**B**), and *OSH2* gene add-back strains (**C**). The strains were cultured overnight, washed with PBS, and then spotted onto YPD or YPD supplemented with miltefosine using 10-fold serial dilutions. After incubating the plates at 30°C for 2 days, photographs were subsequently taken. *CgOSH2*^C193T^ in (**C**) indicates the introduction of the *C. glabrata OSH2* gene with the C193T mutation into the *C. albicans osh2Δ/Δ* strain, and the remaining cases follow accordingly.

Given the absence of genetic manipulation tools and plasmids for *C. glabrata* in our laboratory, we sought to validate our findings using engineered strains of *C. albicans*. We performed gene knockouts targeting the mutated genes that have homologs in *C. albicans*. Spot growth assays revealed that only the *osh2Δ/Δ* strain exhibited resistance to miltefosine (Fig. 3A). To further confirm the phenotype of the *osh2Δ/Δ* strain, we constructed a gene complementation strain and found that resistance to miltefosine was restored in the complemented strain (Fig. 3B). To demonstrate that mutations in the *OSH2* gene of *C. glabrata* are the primary cause of its resistance to miltefosine, we introduced the *C. glabrata OSH2* gene, including its promoter sequence, into the *C. albicans osh2Δ/Δ* strain. Notably, the nonsense mutations within the *OSH2* gene (193C > T and 3177C > A) mediated miltefosine resistance in this strain (Fig. 3C), collectively establishing *OSH2* mutations as a key driver of drug resistance in laboratory-evolved *C. glabrata* populations.

### Multi-omics analysis reveals Osh2 regulates miltefosine resistance by controlling the transport of zymosterol and influencing the ergosterol biosynthesis pathway

**Fig 4:**
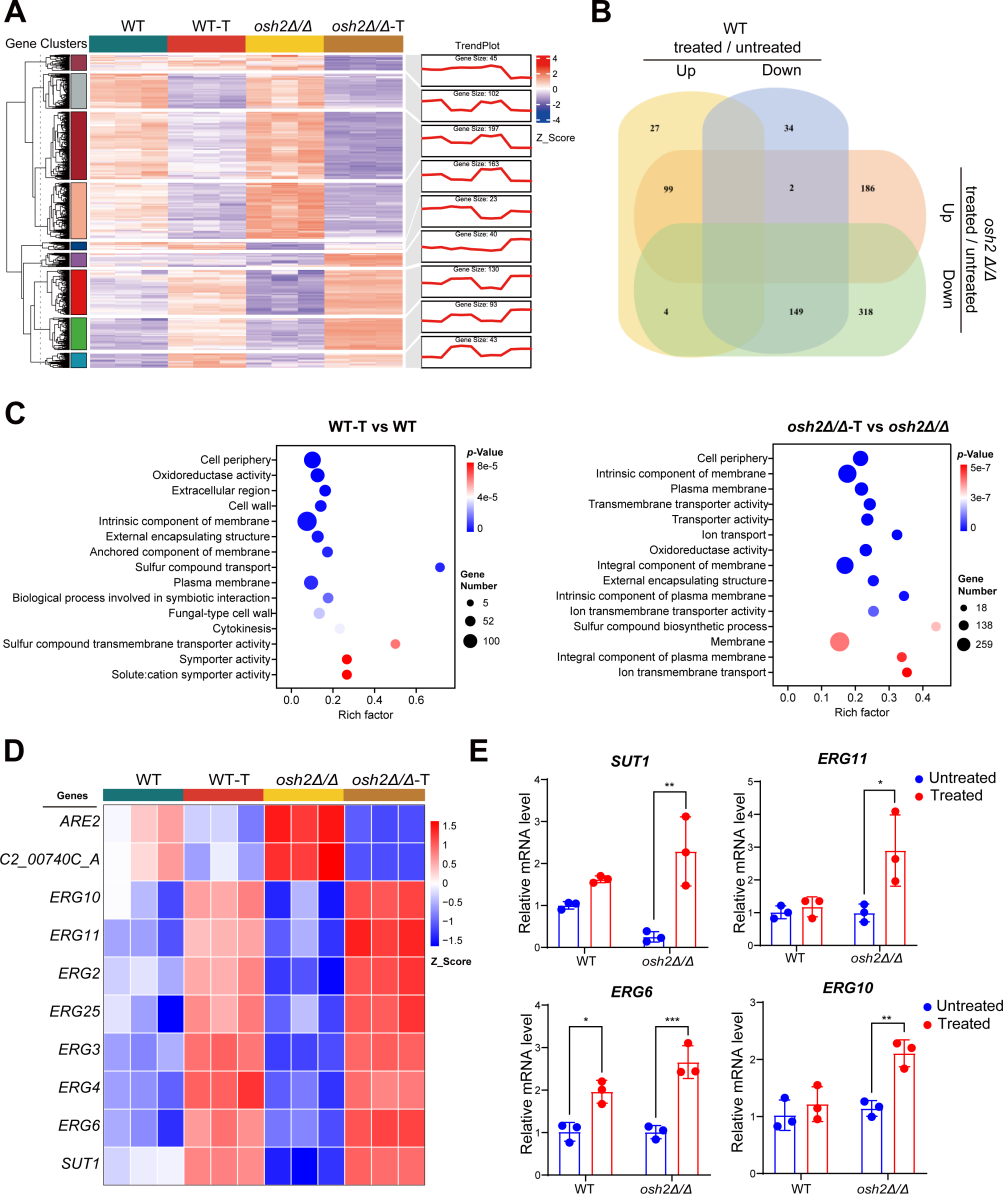
The membrane-related stress response involving ergosterol synthesis genes plays an important role in Osh2-mediated resistance to miltefosine. (**A**) The gene expression heatmap and trend plot show the alterations among different groups. WT-T and *osh2Δ/Δ*-T indicate that the WT and *osh2Δ/Δ* strains were exposed to 2 µg/mL miltefosine for 1 h, respectively. Each group includes three biological replicate samples. (**B**) The Venn diagram illustrates the differentially expressed genes in the WT strain after miltefosine treatment in comparison to the *osh2Δ/Δ* strain. (**C**) GO enrichment analyses were performed on differentially expressed genes affected by miltefosine in both WT and *osh2Δ/Δ* strains. The left panel shows the enrichment analysis results for DEGs in the WT strain, while the right panel displays the results for the *osh2Δ/Δ* strain. The top 15 GO terms are displayed in the bubble plot. (**D**) The heatmap illustrates the expression levels of differentially expressed genes associated with ergosterol biosynthesis across all groups. (**E**) The mRNA levels of the genes *SUT1*, *ERG11*, *ERG6*, and *ERG10* were quantified by RT-qPCR. The data were normalized to the expression of the *ACT1* gene and presented relative to the WT group. The data are expressed as mean ± SD and represent three biological replicates. *P  <  0.05, **P  <  0.01, ***P  <  0.001; by two-way ANOVA with Tukey’s test.

To explore the molecular mechanisms by which Osh2 regulates *C. albicans* resistance to miltefosine, we performed RNA-seq on both wild-type and *osh2Δ/Δ* mutant strains under miltefosine treatment and untreated conditions. After miltefosine treatment, the wild-type strain exhibited 315 differentially expressed genes (DEGs), including 185 downregulated and 130 upregulated genes; whereas the *osh2Δ/Δ* mutant strain showed 758 DEGs, with 287 upregulated and 471 downregulated genes (Fig. 4A and B; Table S2). This suggests that the *osh2Δ/Δ* mutant strain underwent more drastic changes in gene expression patterns after miltefosine treatment. Gene ontology (GO) functional enrichment analysis of the DEGs revealed that both the wild-type and *osh2Δ/Δ* mutant strains had a significant enrichment in various membrane components after miltefosine treatment, with the *osh2Δ/Δ* mutant showing a higher number of enriched genes (Fig. 4C; Table S2). Further analysis indicated that in the *osh2Δ/Δ* mutant strain, the expression of genes related to the ergosterol biosynthesis pathway significantly increased after miltefosine treatment, including *ERG6* and *ERG11* (Fig. 4D). For quantitative real-time PCR (qRT-PCR) validation, we selected four genes (*ERG6*, *ERG10*, *ERG11*, and *SUT1*) spanning different stages of the ergosterol biosynthesis pathway, which were among the most significantly upregulated genes in the RNA-seq data (Fig. 4D). We found that the expression of all these genes in the *osh2Δ/Δ* mutant strain significantly increased after miltefosine treatment, which was consistent with the RNA-seq results, reinforcing the pathway-wide transcriptional response (Fig. 4E).

**Fig 5:**
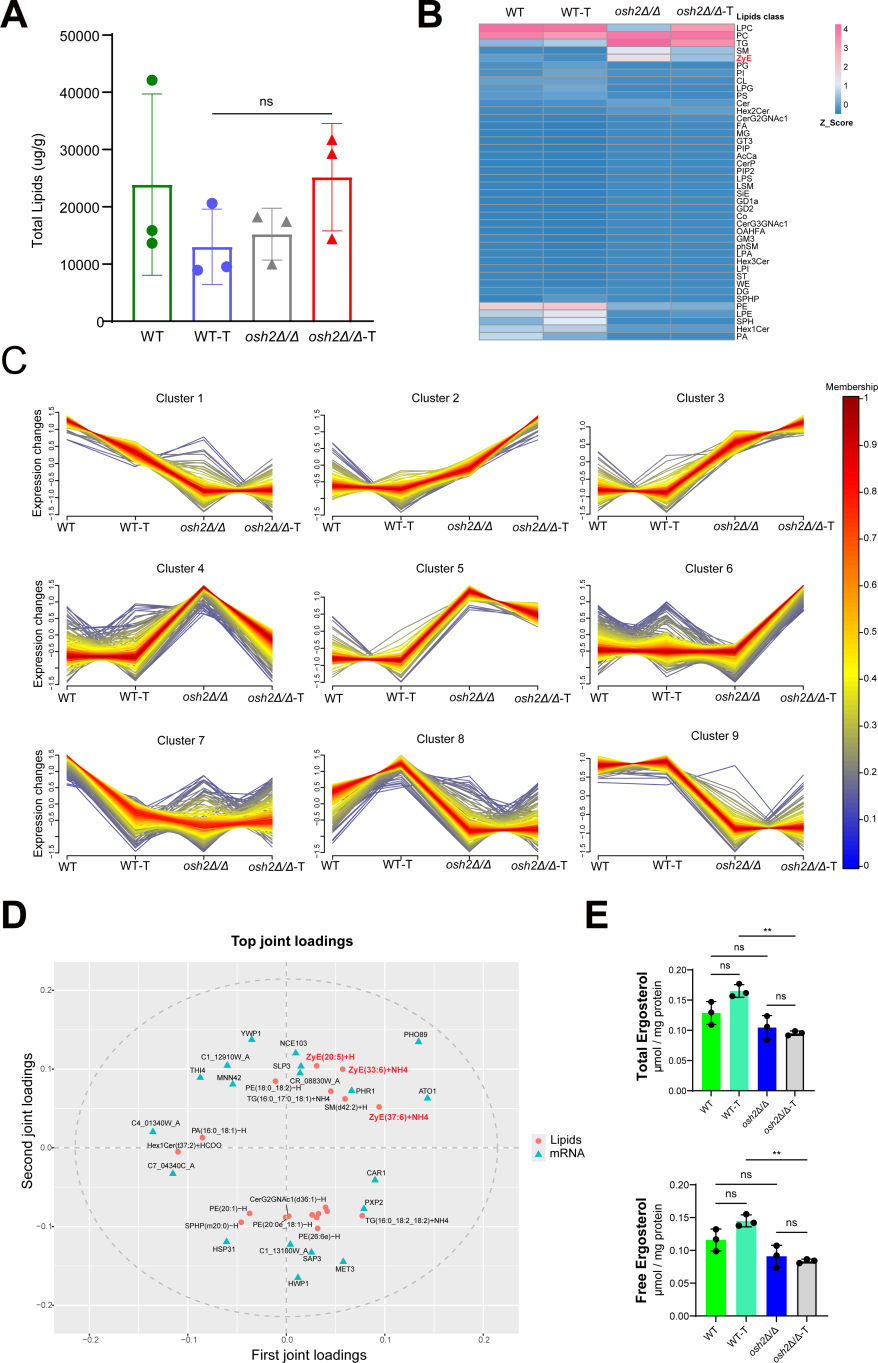
The accumulation of zymosterol plays an important role in miltefosine resistance mediated by Osh2. Based on lipidomics analysis, (**A**) shows the total lipid content of each group of strains, while (**B**) shows the proportion of lipid classes for those groups. (**C**) Trend clustering analysis was employed to elucidate the expression patterns and dynamic changes of all identified lipid molecules across different groups. The fuzzy c-means algorithm was utilized to classify the lipid molecules into distinct clusters based on their expression trends. (**D**) All differentially expressed genes and metabolites were selected to construct an O2PLS model. We initially assessed the correlations and weights of variables in different data groups using the loading plot. This allowed us to identify variables with high correlation and weight. Based on the loading values, we selected the top 20 genes and metabolites with the highest squared loading values in the first two dimensions for visualization in a combined loading plot, highlighting the most associated genes and metabolites. In the plot, each dot represents a lipid metabolite, with larger absolute coordinate values indicating a stronger association with another omic variable. (E) Ergosterol was quantified by liquid chromatography-tandem mass spectrometry (LC-MS/MS). Data are presented as mean ± SD from three independent biological replicates. Statistical significance was determined by one-way ANOVA followed by Tukey's post hoc test (**P < 0.01; ns, not significant).

Based on transcriptome analysis and functional analysis of the Osh2 gene, we hypothesize that Osh2 may regulate lipid metabolism in *Candida* species. We conducted lipidomics analysis on both wild-type and *osh2Δ/Δ* mutant strains under miltefosine treatment and untreated conditions. There was no significant change in the overall lipid content between the wild-type and *osh2Δ/Δ* mutant strains under miltefosine treatment or untreated conditions (Fig. 5A). Comparative lipidomics revealed distinct compositional shifts in the *osh2Δ/Δ* mutant relative to the wild-type strain. Zymosterol constituted 11.9% of total lipids in the mutant vs 2.1% in the wild type under untreated conditions, with this proportional disparity persisting post-miltefosine treatment (5.39% vs 1.0%) (Fig. 5B; Table S3). The mutant additionally displayed elevated triglyceride and stigmasterol ester fractions alongside diminished sphingosine-related lipid classes across both untreated and treated conditions when compared to wild-type counterparts (Fig. 5B; Table S3). Global lipidome-wide clustering analysis of all detected molecules demonstrated marked lipidomic restructuring in the *osh2Δ/Δ* mutant relative to wild type under basal conditions, whereas minimal compositional shifts were observed across strains pre- vs post-miltefosine exposure (Fig. 5C). This topology divergence highlights Osh2-dependent homeostatic regulation of membrane lipid architecture in *C. albicans*.

Comparative lipidomic profiling revealed a striking 478-fold accumulation of ZyE(33:6) + NH4 (a molecular form of zymosterol) in the *osh2Δ/Δ* mutant relative to wild-type controls (Table S4). Integrative multi-omics modeling via O2PLS revealed zymosterol molecules, such as ZyE(20:5) + H, ZyE(33:6) + NH4, and ZyE(37:6)+NH4, as the lipids with high loading values (Fig. 5D). Considering the limitations of untargeted lipidomics profiling in detecting sterols, we conducted targeted LC-MS/MS quantification of ergosterol across matched samples. In wild-type strains, both total and free ergosterol pools showed upward trends following miltefosine treatment, although these changes were not statistically significant (Fig. 5E). In contrast, the *osh2Δ/Δ* mutant exhibited stable ergosterol levels before and after miltefosine exposure yet displayed significant reductions in both total and free ergosterol compared to treated wild-type strains (Fig. 5E).

### Genes involved in the zymosterol metabolic pathway regulate miltefosine sensitivity

**Fig 6:**
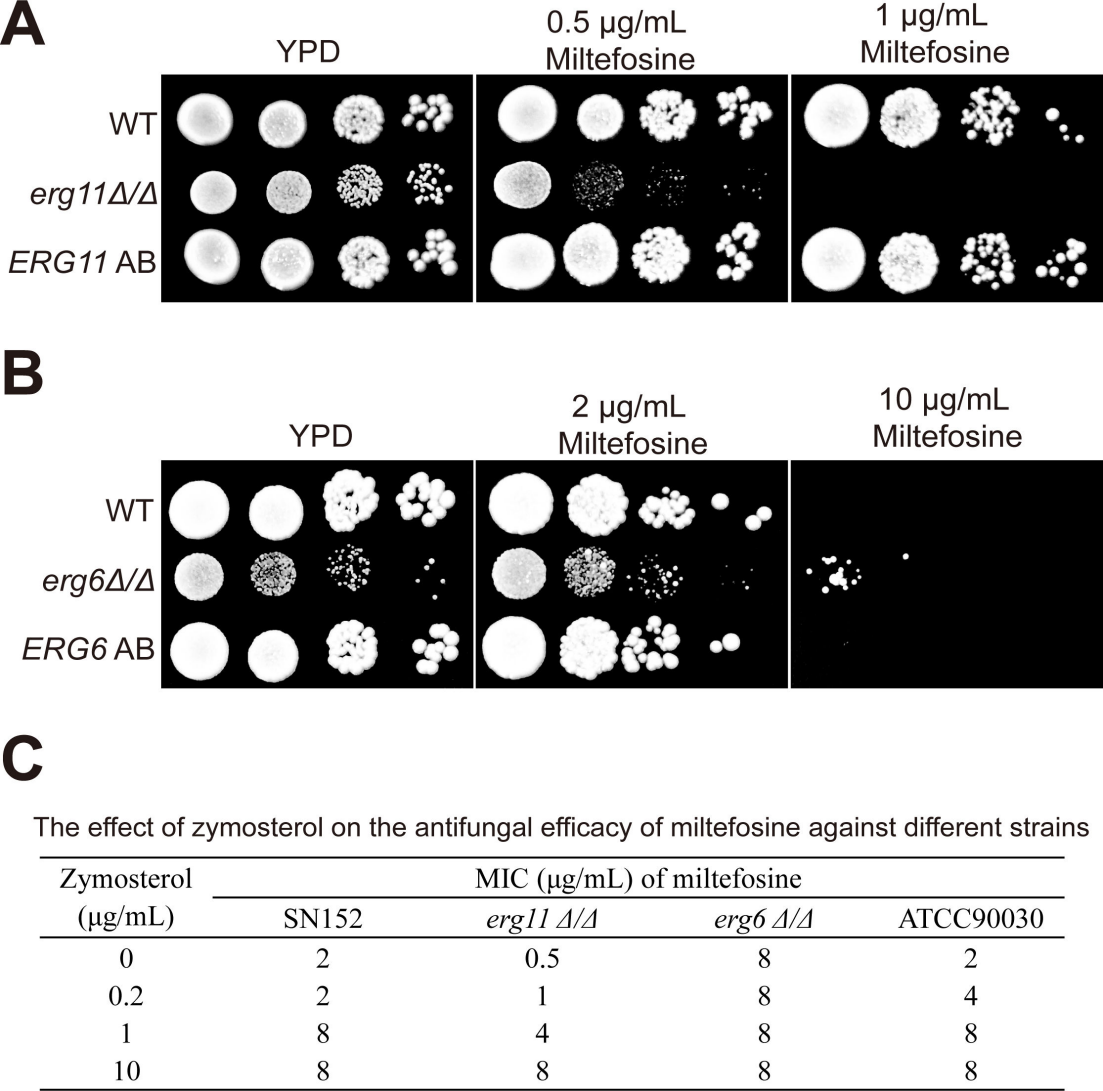
The metabolism of zymosterol affects the susceptibility of the strains to miltefosine. Spot growth assays were conducted on the mutants of gene *ERG11* (**A**) and *ERG6* (**B**). The strains were cultured overnight, washed with PBS, and then spotted onto YPD or YPD supplemented with miltefosine using 10-fold serial dilutions. After incubating the plates at 30°C for 2 days, photographs were subsequently taken. (**C**) The effect of exogenous zymosterol on the antifungal efficacy of miltefosine. The minimum inhibitory concentration of miltefosine for each strain was assessed by incorporating various concentrations of zymosterol into the broth. The experiment was repeated three times, and MIC values were recorded if they were consistent in at least two of the trials.

The zymosterol biosynthesis pathway constitutes a critical branch of fungal ergosterol metabolism, wherein *ERG11*-encoded cytochrome P450 catalyzes zymosterol synthesis, followed by its *ERG6*-dependent conversion to fecosterol. To dissect the role of zymosterol in miltefosine susceptibility, we generated isogenic *erg11Δ/Δ* and *erg6Δ/Δ* mutants through targeted gene disruption. Spot growth assay results revealed contrasting phenotypes: the *erg11Δ/Δ* mutant displayed miltefosine hypersensitivity (Fig. 6A), whereas *erg6Δ/Δ* strains exhibited intrinsic resistance (Fig. 6B), consistent with zymosterol accumulation. Exogenous zymosterol supplementation dose dependently increased miltefosine MIC values across tested strains, with the exception of the *erg6Δ/Δ* mutant. Notably, the *erg11Δ/Δ* mutant showed a 16-fold MIC elevation (from 0.5 to 8 µg/mL) under maximal zymosterol induction, paralleled by a 4-fold increase in *C. albicans* SN152 and *C. glabrata* ATCC90030 (Fig. 6C). These orthogonal approaches demonstrate that zymosterol homeostasis critically modulates membrane susceptibility to miltefosine through sterol structural accommodation mechanisms.

## DISCUSSION

**Fig 7:**
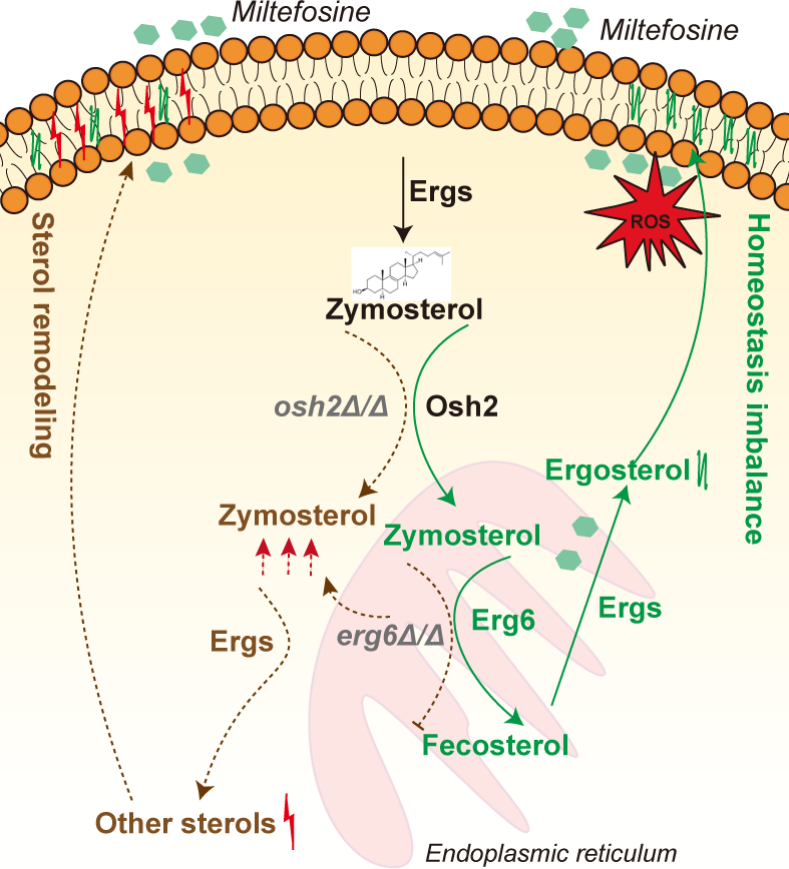
The schematic of the accumulation of zymosterol metabolism in mediating the resistance of *Candida* species to miltefosine. In the ergosterol biosynthesis pathway, zymosterol is synthesized through sequential enzymatic reactions (e.g., catalyzed by Erg10 and Erg11) and transported to the endoplasmic reticulum (ER) by Osh2. Within the ER, zymosterol is converted to fecosterol by Erg6, ultimately yielding ergosterol for membrane homeostasis. Miltefosine disrupts membrane integrity by inducing ROS. Our study reveals that Osh2 dysfunction prevents zymosterol transport to the ER, leading to its intracellular accumulation. Similarly, *ERG6* deletion blocks zymosterol-to-fecosterol conversion, causing zymosterol buildup. Zymosterol accumulation triggers alternative sterol remodeling, restoring membrane stability under miltefosine pressure. Exogenous zymosterol supplementation phenocopies this resistance, confirming its central role in modulating miltefosine susceptibility.

Our study uncovers a novel mechanism of miltefosine resistance in *Candida* species, driven by nonsense mutations in the *OSH2* gene and the subsequent accumulation of zymosterol. In the ergosterol biosynthesis pathway, zymosterol is synthesized by sequential enzymatic reactions, then transported to the endoplasmic reticulum by Osh2, where it is converted to fecosterol by Erg6, ultimately producing ergosterol for membrane stability (27). Our study demonstrates that disruption of either Osh2-mediated transport or Erg6-dependent conversion leads to intracellular zymosterol accumulation. Beyond its role as a metabolic intermediate, zymosterol accumulation remodels membrane sterol composition and confers miltefosine resistance. Exogenous zymosterol supplementation recapitulates this resistant phenotype, confirming sterol homeostasis as a critical determinant of miltefosine susceptibility. A schematic is shown in Fig. 7.

In this study, we successfully induced miltefosine-resistant strains of *C. glabrata* through stepwise exposure to increasing drug concentrations, confirming the strain’s capability to adapt through genetic mutations. For antifungal drugs targeting the ergosterol biosynthesis pathway, fungal pathogens often evolve resistance by acquiring mutations in genes related to this pathway, thereby modifying sterol synthesis to produce lipids that stabilize the cell membrane (28–30). Under miltefosine pressure, *C. glabrata* naturally developed nonsense mutations in *OSH2*, enabling survival by altering the ergosterol biosynthesis pathway. This observation is consistent with previous studies, showing that exogenous addition of ergosterol reduces the antifungal efficacy of miltefosine (17, 31). Similarly, we found that supplementing with zymosterol, an intermediate in the ergosterol biosynthesis pathway, also diminished miltefosine’s activity. Collectively, these results suggest that the ergosterol biosynthesis pathway may be a key target of miltefosine. However, further evidence is needed to confirm this hypothesis. Future studies could employ direct binding assays, genetic complementation, lipidomic profiling, and structural biology approaches to validate this mechanism.

While our findings position miltefosine as a potential ergosterol biosynthesis pathway inhibitor, whole-genome sequencing of laboratory-evolved miltefosine-resistant *C. glabrata* strains identified no missense mutations in classical ergosterol biosynthesis genes (Table S1). This contrasts sharply with established resistance mechanisms: (i) azole resistance, which is primarily mediated by gain-of-function mutations in the transcription factor Pdr1 that upregulate efflux pumps or by missense mutations in *ERG11*, and (ii) polyene resistance, which is typically caused by mutations in ergosterol biosynthetic enzymes (e.g., Erg6) (32–34). Importantly, we demonstrate that *C. glabrata* evolves miltefosine resistance specifically through loss-of-function mutations in the sterol transporter Osh2, thereby revealing a novel adaptive strategy. Notably, neither the laboratory-evolved *C. glabrata* mutants nor the engineered *C. albicans osh2Δ/Δ* strains displayed cross-resistance to fluconazole or amphotericin B (Table S6), confirming that Osh2-mediated zymosterol accumulation represents a miltefosine-specific resistance mechanism distinct from classical ergosterol pathway alterations.

Moreover, this study underscores the critical role of lipid metabolism pathways, particularly sterol biosynthesis, in mediating antifungal resistance. Sterol metabolism, particularly ergosterol biosynthesis, is a cornerstone of fungal cell membrane integrity and a major target for antifungal therapies (35). For example, azoles inhibit ergosterol synthesis, disrupting membrane architecture. In response, fungal pathogens adapt by modifying the ergosterol biosynthetic pathway (36). Alterations in ergosterol content and intermediate sterol profiles are key strategies employed by fungi to maintain membrane fluidity under drug pressure (33, 37, 38). A notable example is observed in the *Mucor* species complex, where loss of Erg6a function redirects sterol biosynthesis toward the production of cholesta-type sterols, thereby preserving membrane permeability and conferring resistance to amphotericin B (36). Similarly, our results demonstrate that the *osh2Δ/Δ* strain survives miltefosine stress by accumulating zymosterol and reducing ergosterol content, thereby modifying the composition of the cell membrane. *ERG11* encodes lanosterol demethylase, a key enzyme in ergosterol biosynthesis, whose substrate is subsequently converted into zymosterol (27). Osh2 regulates the transport of zymosterol to the endoplasmic reticulum, where Erg6 catalyzes its conversion to fecosterol (39, 40). Here, we demonstrate that disruption of the *ERG11* gene increases susceptibility to miltefosine, while deletion of *ERG6* confers resistance to the drug. However, *erg6Δ/Δ* strains exhibit severe growth defects, highlighting the fitness cost associated with this adaptation. Interestingly, *osh2Δ/Δ* strains exhibit no significant growth impairment or fitness costs under standard and stress conditions (Fig. S2) while still conferring robust miltefosine resistance. This suggests that *C. glabrata* preferentially mutates the *OSH2* gene to survive miltefosine stress, as it minimizes fitness costs while achieving resistance through zymosterol accumulation. This metabolic plasticity highlights the remarkable ability of fungi to circumvent targeted antifungal inhibition through sterol pathway remodeling.

Despite these insights, our study has several limitations. First, we were unable to directly verify whether miltefosine binds to the Osh2 protein or zymosterol, which would provide more definitive evidence for the proposed mechanism. Additionally, due to technical limitations in our laboratory, genetic manipulation of the *OSH2* gene in *C. glabrata* was not feasible, necessitating the use of *C. albicans* as an alternative model. However, we demonstrated functional conservation between the two species by complementing the *C. albicans osh2Δ/Δ* strain with the *C. glabrata OSH2* gene and confirming that the nonsense mutation in *C. glabrata OSH2* conferred a similar resistance phenotype in *C. albicans*. These results support the relevance of our findings to *C. glabrata* despite the use of a surrogate model. Future work should focus on identifying miltefosine’s direct targets and exploring structural interactions. Furthermore, the development of novel antifungal drugs targeting zymosterol transport or other components of the ergosterol biosynthesis pathway may hold significant clinical promise. By addressing these questions, we can deepen our understanding of miltefosine’s mechanism of action and develop more effective strategies to combat antifungal resistance.

## MATERIALS AND METHODS

### Strains, media, and chemicals

The strains *C. albicans* SC5314, *C. glabrata* ATCC90030, *C. tropicalis* ATCC750, *C. krusei* ATCC 6258, and *C. auris* CBS12766 were utilized to induce resistance to miltefosine, thereby generating miltefosine-resistant mutants for further study. Strains and plasmids used in this study are listed in Table S5. All mutants were generated in the *C. albicans* SN152 auxotrophic background using homologous recombination with *HIS1* (first allele) and *LEU2* (second allele) markers, whereas gene complementation was performed employing the *ARG4* marker, as previously described (41). Primers used for strain construction are listed in Table S6.

To generate the *osh2Δ/Δ* mutant in *C. albicans* SN152, we employed a sequential homologous recombination strategy. For the first allele knockout, the 5′ and 3′ flanking regions of *OSH2* were amplified using primer pairs Osh2W1/W3 and Osh2W4/W6, respectively. These fragments were then fused with the *HIS1* selectable marker (amplified with universal primers W2/W5) via overlap PCR, and the resulting construct was transformed into SN152 competent cells. Primary transformants were selected on SC-His plates and verified by PCR using flanking primer sets Osh2UP-F1/R1 (upstream integration) and Osh2DW-F1/R1 (downstream integration). The second allele was deleted similarly, except the 5′ flank was amplified with Osh2W1/W7 to generate a fragment of distinct length, thereby reducing the probability of ectopic integration. These fragments were fused with the *LEU2* marker and transformed into the heterozygous mutant. Double knockouts were selected on SC-His-Leu plates and verified using primer sets Osh2UP-F2/R2 and Osh2DW-F2/R2, with homozygous deletion confirmed by PCR amplification failure using ORF-specific primers Osh2ORF-F/R. The construction of other mutants, such as *hgt6Δ/Δ*, followed similar procedures. The *erg11Δ/Δ* mutant was obtained from our laboratory collection, as this strain has been previously characterized and published (42).

For genetic complementation, the full-length *OSH2* expression cassette, including promoter and terminator regions, was amplified using primers OSH2AB-F/R and cloned into the ARG4-marked plasmid pCB197 (18). The construct was linearized with KpnI/ApaI restriction enzymes and transformed into the *osh2Δ/Δ* mutant. Transformants were selected on SC-Arg plates and verified by ORF-specific PCR. Other gene complementations were performed using the same method.

To ensure proper translation of *C. glabrata OSH2* in *C. albicans*, we codon optimized the entire coding sequence by replacing all CTG codons with TTG to prevent mistranslation in the CTG-clade *C. albicans* while preserving the amino acid sequence. The optimized gene fragment, including the native promoter and terminator regions, was commercially synthesized by Tsingke Biotechnology Co., Ltd. (Nanjing, China). It was then cloned into the pCB197 plasmid using the Trelief Seamless Cloning Kit and transformed into the *osh2Δ/Δ* mutant after linearization with KpnI/ApaI. Successful complementation was confirmed by PCR verification using primers CgOsh2ORF-F/R.

Strains are commonly pre-incubated in YPD medium at 30°C with shaking at 220 rpm. RPMI 1640 culture medium was used for MIC determination. Miltefosine was obtained from Sigma-Aldrich. Fluconazole and amphotericin B were obtained from Selleck. Zymosterol was obtained from MedChemExpress.

### MIC determination

The minimum inhibitory concentration of miltefosine against clinically relevant *Candida* species was assessed using the broth microdilution method in strict accordance with Clinical and Laboratory Standards Institute M27-A4 guidelines. MIC endpoints were defined as the lowest concentration demonstrating complete growth inhibition after 24 h of incubation at 35°C under visual examination. All experiments were conducted in triplicate biological replicates.

### Development of miltefosine resistance in *Candida* species using a concentration gradient protocol

The resistance induction process for the five *Candida* strains (*C. albicans* SC5314, *C. glabrata* ATCC90030, *C. tropicalis* ATCC750, *C. krusei* ATCC6258, and *C. auris* CBS12766) was conducted as follows: yeast cells were pre-cultured in YPD broth overnight at 30°C with 220 rpm. For initial exposure, cultures were inoculated (OD_600_ = 0.2) into fresh YPD containing miltefosine at 0.5× MIC and incubated for 48 h under identical conditions. Following this, cells were transferred to drug-free YPD for 24 h to complete one full passage cycle. Drug pressure escalation was triggered only when the final OD_600_ in the miltefosine-containing phase exceeded 1.0, at which point the subsequent passage utilized doubled drug concentrations (e.g., 1× MIC, 2× MIC). If growth failed to reach OD_600_ >1.0, the same concentration was maintained. This iterative process continued for 30 passages, with aliquots cryopreserved at 10-passage intervals for phenotypic validation. MIC fold increases were calculated relative to parental strains.

### Spot dilution assays

All strains were cultured overnight in YPD broth at 30°C with 200 rpm shaking. The cell suspensions were then centrifuged, washed with PBS, and standardized to OD_600_ = 0.1. Tenfold serial dilutions (from 10⁰ to 10⁻³) were prepared in PBS, and 5 µL aliquots of each dilution were spot inoculated onto YPD agar plates containing varying concentrations of miltefosine. The plates were incubated at 30°C for 24–48 h, after which images were captured.

### Whole-genome sequencing

Whole-genome sequencing was performed with distinct strategies: *de novo* sequencing was conducted for the ATCC750 strain using the Illumina NovaSeq 6000 platform (paired-end 150 bp), while three mutant strains underwent resequencing against the *de novo*-assembled reference genome. Genomic DNA was extracted via phenol-chloroform methodology, and library preparation was executed with Illumina TruSeq DNA PCR-free kits. Libraries passing quality thresholds (Agilent Bioanalyzer HS DNA Kit, DV200 >80%) were normalized, denatured with 0.1 N NaOH, and clustered at optimal densities. Raw sequencing data underwent FASTP-based quality trimming (Q20 threshold, adapter removal) prior to assembly. For *de novo* reconstruction, Falcon (v1.8.1) and CANU (v2.2) generated contigs (N50 >100 kb), polished iteratively with Pilon (v1.18) using Illumina reads. Resequencing data were aligned via BWA-MEM (v0.7.17), followed by variant calling with GATK HaplotypeCaller (v4.2.6.1). Structural variations, including CNVs, were detected through CNVnator (v0.4.1) with 1 kb bin resolution. All experimental and computational workflows were implemented under ISO-certified protocols by Shanghai Personal Biotechnology Co., Ltd.

### Sequencing of the *CAGL0K01749g* gene in *C. glabrata*

Genomic DNA was extracted from the target strains, and the *CAGL0K01749g* gene was amplified using the primers Osh2Seq-F and Osh2Seq-R. The purified PCR products were subjected to bidirectional Sanger sequencing at Tsingke Biotechnology Co., Ltd. (Nanjing, China). The resulting sequences were aligned with the reference sequence of *CAGL0K01749g* in the *Candida* Genome Database for comparative analysis.

### RNA-seq and bioinformatics analysis

The strains SN152 and *osh2Δ/Δ* were incubated at 30°C for 1 h, in the presence and absence of miltefosine. Subsequently, RNA-seq analysis was performed as previously described (18). In brief, total RNA was isolated using the Quick-RNA Microprep Kit (ZYMO Research) with on-column DNase I digestion, followed by mRNA enrichment via poly(A) selection. Sequencing libraries were prepared following the Illumina Stranded mRNA protocol and paired-end sequenced (2 × 150 bp) on the Illumina NovaSeq 6000 platform through Shanghai Personal Biotechnology Co., Ltd., achieving >20 million reads per sample. Processed reads were aligned to the *C. albicans* SC5314 reference genome (GenBank: GCF_000182965.3). Gene expression quantification was performed and normalized as fragments per kilobase per million mapped reads. Differential expression analysis was performed using DESeq2 (version 1.34.0). Genes were considered significantly differentially expressed if they exhibited a fold change of ≥1.5 and a *P*-value of <0.05. Three independent biological replicates were analyzed.

### Lipidomics determination and analysis

Lipidomic profiling was performed on *C. albicans* strains SN152 and *osh2Δ/Δ* following 1 h exposure to 2 µg/mL miltefosine or untreated controls. Cell pellets were homogenized with 200 µL of water and 20 µL of internal lipid standards (Avanti Polar Lipids). Lipid extraction was then conducted using a methyl tert-butyl ether (MTBE)/methanol protocol (800 µL MTBE +240 µL methanol), followed by vortexing, ice-bath sonication for 20 min, and phase separation (14,000 × *g*, 10°C, 15 min). The dried lipid extracts were reconstituted in 90% isopropanol/acetonitrile for subsequent UHPLC-MS/MS analysis. Chromatographic separation was achieved using a Nexera LC-30A system (Shimadzu) equipped with a C18 column at 45°C. The mobile phases consisted of (i) 60% acetonitrile with 0.1% formic acid and 0.1 mM ammonium formate, and (ii) 90% isopropanol with 0.1% formic acid and 0.1 mM ammonium formate. The elution was performed at a flow rate of 300 µL/min using a gradient program: 30% B (0–2 min), increasing to 100% B (2–25 min), and holding at 100% B (25–35 min). Mass spectrometry was conducted on a Q Exactive HF-X mass spectrometer (Thermo Scientific) in data-dependent acquisition mode. Lipid identification and quantification were performed using LipidSearch v4.2 software (Thermo Scientific). Differential lipids were identified using orthogonal partial least squares-discriminant analysis (VIP >1) and Student’s *t*-test (*P* < 0.05).

### Quantitative real-time PCR

Yeast cells in the logarithmic growth phase were harvested, and total RNA was isolated using the Quick-RNA Microprep Kit (ZYMO Research, USA). Subsequently, cDNA was synthesized from the extracted RNA using the PrimeScript RT Reagent Kit (TaKaRa, Tokyo, Japan). qRT-PCR was performed using the SYBR Premix Ex Taq II (Tli RNaseH Plus) Kit with the primers detailed in Table S6. The relative expression levels of the target genes were determined using the 2^−ΔΔCt^ method and normalized to the reference gene *ACT1*. Each gene was analyzed in three independent biological replicates.

### Ergosterol detection

Lipids were extracted using a modified Bligh-Dyer protocol. Cell pellets were homogenized in 750 µL of chloroform/methanol/MilliQ water (3:6:1, [vol/vol/vol]) and incubated at 1,500 rpm for 1 h at 4°C. Phase separation was induced by sequentially adding 350 µL of deionized water and 250 µL of chloroform, followed by centrifugation to collect the lipid-containing organic phase. The extraction was repeated with 450 µL of chloroform, and the combined organic phases were dried under vacuum (SpeedVac, OH mode). The residual aqueous phases and pellets were vacuum dried for subsequent protein quantification. Dried lipid extracts were reconstituted in 200 µL of 90% isopropanol/acetonitrile and analyzed using a Jasper HPLC system equipped with a C18 column coupled to a Sciex 4500 MD mass spectrometer in atmospheric pressure chemical ionization mode. Deuterated internal standards (d6-cholesterol and d6-C18:0 cholesteryl ester; CDN isotopes) were used for absolute quantification. Final values were normalized to total protein content (μmol ergosterol/mg protein) to account for biological variability.

## Data Availability

All data that support the findings of this study are available in the paper, as well as in the supplementary information. The *CAGL0K01749g* gene sequences have been submitted to GenBank with accession numbers ranging from PV189419 to PV189421. The whole-genome sequencing data have been deposited in the NCBI Sequence Read Archive under the accession number PRJNA1226902. Additionally, the RNA-Seq data are accessible in the NCBI Sequence Read Archive with the accession number PRJNA1227393. Furthermore, the untargeted lipidomic data have been deposited to the EMBL-EBI MetaboLights database with the identifier MTBLS12270.

## SUPPLEMENTAL MATERIAL

Fig. S1 aac.00427-25-s0001.tif The Sanger sequencing chromatogram.

Fig. S2 aac.00427-25-s0002.tif Loss of OSH2 has no growth defect or fitness cost.

Supplemental material aac.00427-25-s0003.docx Legends for all Supplemental figures and tables.

Table S1 aac.00427-25-s0004.xlsx Missense mutations (SNPs) and Indels.

Table S2 aac.00427-25-s0005.xlsx Differentially expressed genes and related statistical analysis.

Table S3 aac.00427-25-s0006.xlsx Lipids class percentages.

Table S4 aac.00427-25-s0007.xlsx Differential lipids analysis.

Table S5 aac.00427-25-s0008.xlsx Strains and plasmids used in this study.

Table S6 aac.00427-25-s0009.xlsx Primers used in this study.

ASM does not own the copyrights to Supplemental Material that may be linked to, or accessed through, an article. The authors have granted ASM a non-exclusive, world-wide license to publish the Supplemental Material files. Please contact the corresponding author directly for reuse.
