# Supplementary figures and images for: Osh2 mediates Candida species resistance to miltefosine by regulating zymosterol accumulation
Source: Antimicrob Agents Chemother. 2025 Jul 23;69(9):e00427-25. doi: 10.1128/aac.00427-25 (PMC12406662; doi:10.1128/aac.00427-25)

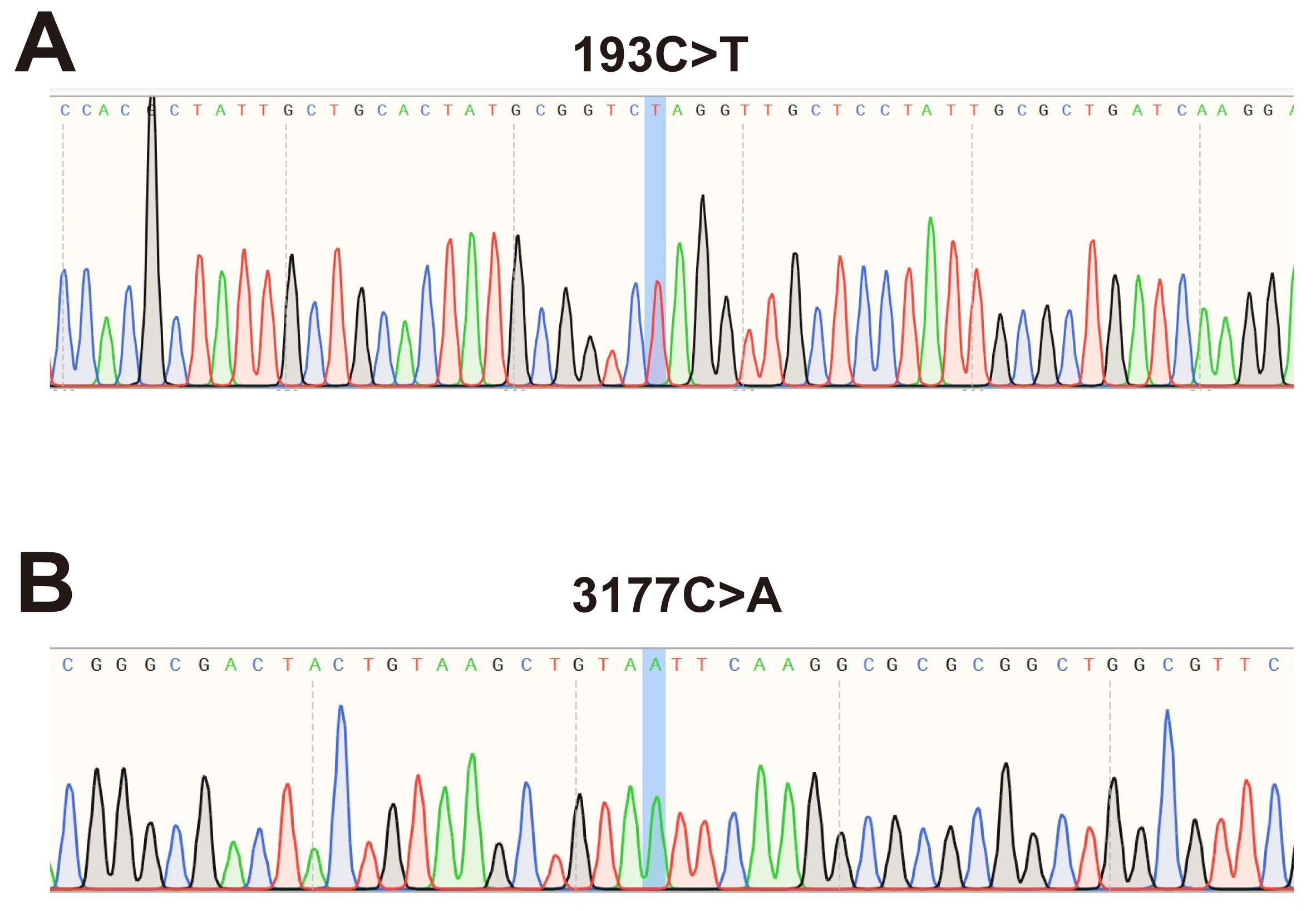

Supplement: Fig. S1 — The Sanger sequencing chromatogram. [file aac.00427-25-s0001.tif]

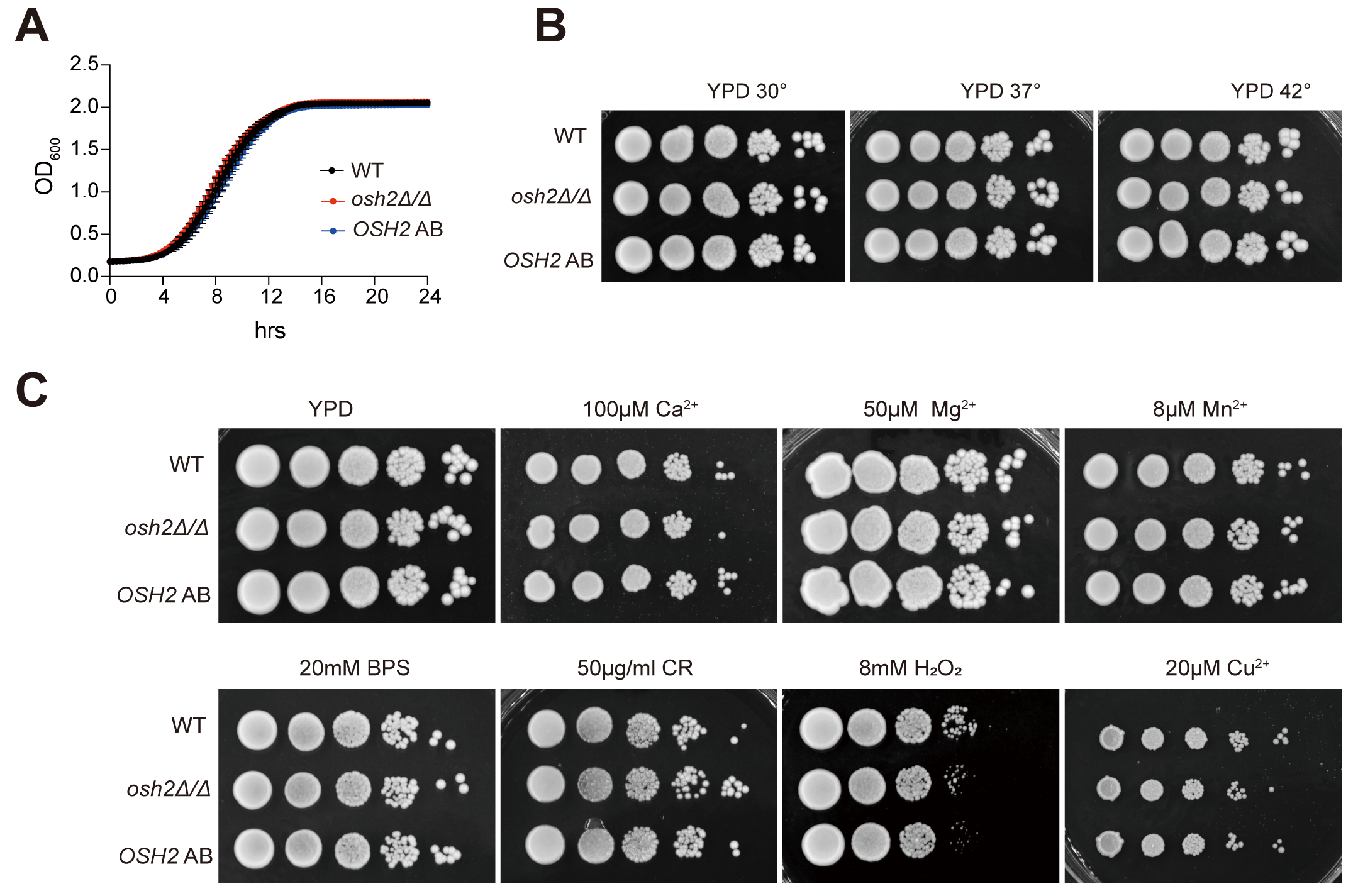

Supplement: Fig. S2 — Loss of OSH2 has no growth defect or fitness cost. [file aac.00427-25-s0002.tif]
