# Supplementary material for: Osh2 mediates Candida species resistance to miltefosine by regulating zymosterol accumulation
Source: Antimicrob Agents Chemother. 2025 Jul 23;69(9):e00427-25. doi: 10.1128/aac.00427-25 (PMC12406662; doi:10.1128/aac.00427-25)
Supplement: Supplemental material — Legends for all Supplemental figures and tables. [file aac.00427-25-s0003.docx]

**Supplemental legends:**

Figure S1. The Sanger sequencing chromatogram. (A) The chromatogram revealed a C-to-T transition at position 193, resulting in a premature stop codon (nonsense mutation); (B) The chromatogram revealed a C-to-A transition at position 3177, resulting in a premature stop codon (nonsense mutation).

Figure S2. Loss of *OSH2* has no growth defect or fitness cost. (A) Growth curve analysis of WT, *osh2Δ/Δ* and OSH2AB strains in YPD liquid medium at 30 ̊°C by measuring OD_600_ at the indicated time points. (B) WT, *osh2Δ/Δ* and OSH2AB strains were spotted with tenfold serial dilutions onto YPD and grown for 2 d at 30 °C, 37 °C and 42 °C. (C) WT, *osh2Δ/Δ* and OSH2AB strains were spotted with tenfold serial dilutions onto YPD or YPD supplemented with indicated stress and grown for 2 d at 30 °C.

Table S1. Missense mutations (SNPs) and indels.

Table S2. Differentially expressed genes and related statistical analysis.

Table S3. Lipids class percentages.

Table S4. Differential lipids analysis.

Table S5. Strains and plasmids used in this study.

Table S6. Primers used in this study.
